# Envisioning the Future of Mosaic Landscapes: Actor Perceptions in a Mixed Cocoa/Oil-Palm Area in Ghana

**DOI:** 10.1007/s00267-020-01368-4

**Published:** 2020-10-15

**Authors:** Kwabena O. Asubonteng, Mirjam A. F. Ros-Tonen, Isa Baud, Karin Pfeffer

**Affiliations:** 1 Amsterdam Institute for Social Science Research (AISSR), University of Amsterdam, Nieuwe Achtergracht 166, 1018 VW Amsterdam, The Netherlands; 2 Faculty of Geo-Information Science and Earth Observation (ITC), University of Twente, P.O. Box 217, 7500 AE Enschede, The Netherlands

**Keywords:** Landscape integration–segregation, Smallholders, Participatory spatial scenario building, Landscape approach, Tree crops, Ghana

## Abstract

The future and benefits of mosaic landscapes have been a source of scientific and societal concern due to increasing population growth, climate change, urbanization, and expanding agricultural commodities. There is a growing call for integrated landscape approaches in which landscape actors discuss trade-offs between different land uses with a view to reaching a negotiated decision on the allocation of land uses. Yet, the operationalization of such approaches is still in its infancy, and integrated methodologies to visualize actors’ landscape visions are still scarce. This study therefore presents a participatory spatial scenario-building methodology that uncovers local perceptions of landscape dynamics and needed actions in a mixed cocoa-oil-palm landscape in Ghana’s Eastern Region. The methodology visualizes landscape actors’ perceived plausible changes and desired future landscapes, and is designed to trigger discussions on actions needed to achieve these desired futures. Findings show that farmers and institutional actors are aware of their landscapes with future preferences coming close to actual landscape composition and spatial configuration, and that—contrary to common assumptions—only those in the oil-palm-dominated landscape who already experienced the drawbacks of increasing landscape homogenization desire a mosaic landscape. The paper concludes that the collective mapping process makes actors aware of challenges at landscape level and increases farmers’ negotiation power through active engagement in the process and visualization of their knowledge and visions. Application of the methodology requires dedicated funding, political will, and capacity to apply it as an ongoing process, as well as monitoring feedback loops.

## Introduction

Mosaic landscapes^1^ provide ecosystem services relevant for biodiversity conservation, carbon storage, rural livelihoods, and the sustainable production of food and other products, while enhancing the connectivity needed for the movement of animals and maintenance of natural processes (Van Noordwijk et al. 2012; Kremen and Merenlender 2018). However, concerns exist about their degradation and increasing homogenization due to population growth, urbanization, climate change, and expanding agricultural commodities (van Vliet et al. 2012; Sayer et al. 2013; Benefoh et al. 2018; Asubonteng et al. 2020). This implies a development toward more segregated and specialized landscapes with less variety of ecosystem services, at the cost of the resilience of landscapes and the people depending on them (Tscharntke et al. 2005; Castella et al. 2013; Grass et al. 2020).

In a lively “sparing/sharing” debate, sparing proponents (e.g., Phalan et al. 2011; Cannon et al. 2019) provide evidence and arguments in favor of segregated landscapes where protected areas are set aside from intensive food and commercial crop monocultures. In this way, they aim to optimize both biodiversity conservation and production outcomes. Sharing advocates demonstrate that (agro-)biodiversity is best maintained by integrating multiple land uses in mosaic landscapes (e.g., Perfecto and Vandermeer 2010; Fischer et al. 2017). They often implicitly or explicitly assume that “sharing”—or “wildlife friendly farming” as Fischer et al. (2008) call it—is the preferred strategy in smallholder-dominated landscapes (e.g., Tscharntke et al. 2011; Kremen 2015). However, there is little scientific evidence of smallholder farmers’ preferences for sharing or sparing. Also, a more spatially explicit strand of literature that frames the choices in terms of integration (“sharing”) and segregation (“sparing”) (e.g., Van Noordwijk et al. 2012; Dewi et al. 2013) barely considers actors’ preferences, with the exception of Jiren et al. (2018) and Karner et al. (2019). Consequently, policy instruments that build on smallholders’ views are scarce (Tscharntke et al. 2012).

However, with the emergence of integrated landscape approaches (ILAs), there is a growing call for stakeholder inclusion in landscape decision-making. These approaches argue for integrated and more inclusive forms of landscape governance, based on multiactor and multisector negotiations of trade-offs between different land uses (Sayer et al. 2013; Freeman et al. 2015; Torquebiau 2015; Reed et al. 2016; Ros-Tonen et al. 2018 and other articles in Environmental Management 62(1)). However, the actual implementation of ILAs is still in its infancy and the “persistent gap between theory and application” (Reed et al. 2020, 2013) results in a debate that is still distant from actual landscape realities (Torquebiau 2015; Bürgi et al. 2017; Ros-Tonen et al. 2018). Moreover, such approaches are fraught with power differences and associated questions such as who actually decides on the directions to be taken, based on what motivations, in whose interests, and at what opportunity costs (Clay 2016; Arts et al. 2017; Ros-Tonen et al. 2018). Hence, a crucial step in the operationalization and implementation of landscape approaches is to uncover local perceptions and preferences regarding future landscape scenarios. Yet, studies revealing actors’ visions on the current and future landscape are scarce, with the work of Pfund et al. (2011) among the exceptions.

Hence, this paper addresses knowledge gaps related to (i) the lack of insight into smallholder farmers’ opinions in the sparing/sharing debate, (ii) the lack of methodologies in ILAs to uncover landscape actors’ visions on the desired landscape, and (iii) the still limited attention to landscape configuration in the participatory spatial analysis of landscape dynamics. To do so, it innovatively combines focus group discussions, participatory mapping, and scenario building into a participatory spatial scenario-building methodology to uncover perceptions of landscape dynamics and needed actions in a mixed cocoa-oil-palm landscape in Ghana’s Eastern Region.

**Fig. 1:**
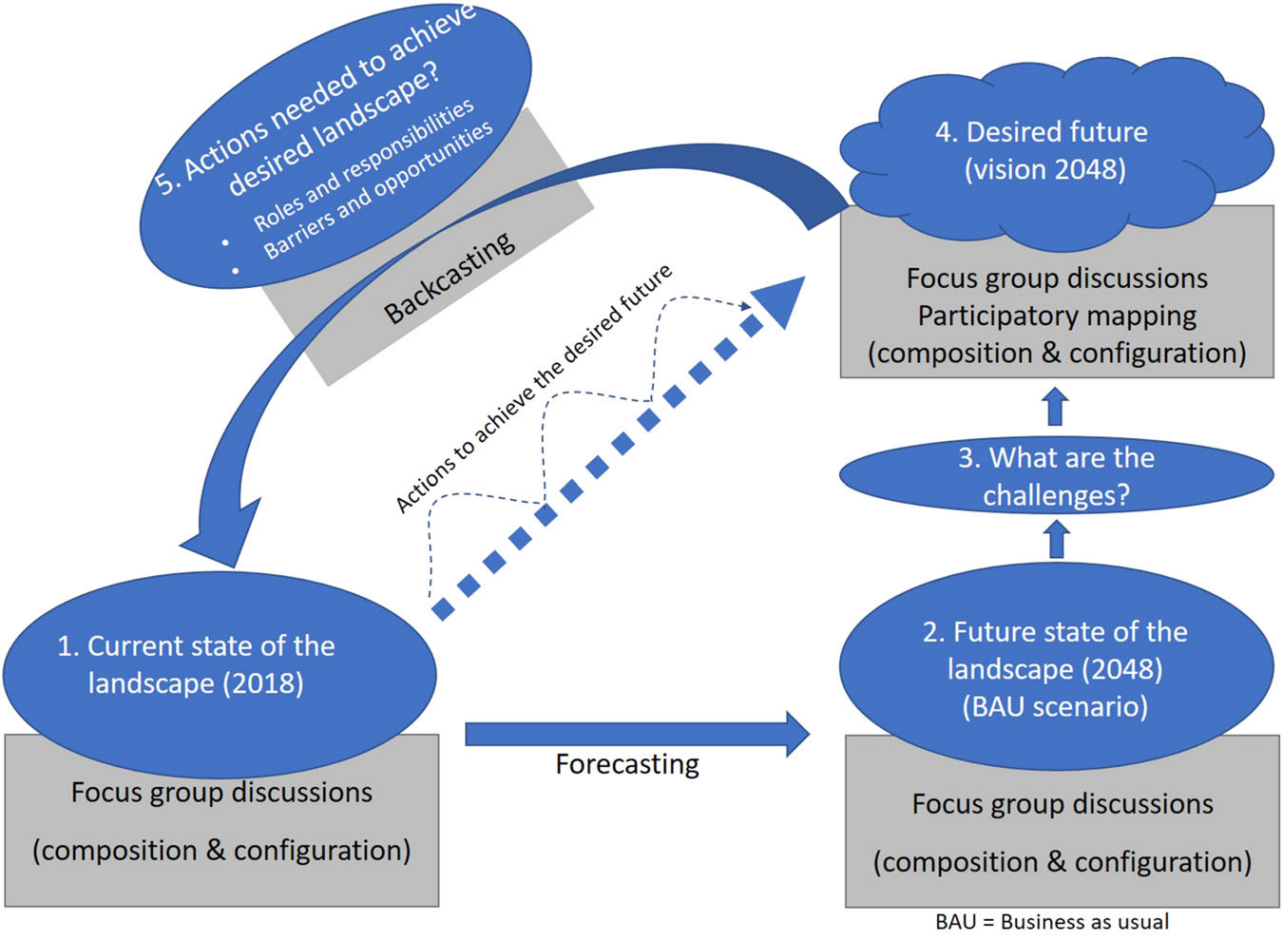
The participatory spatial scenario building methodology in five steps

Following the steps of a scenario-building approach (Fig. 1), it asks smallholder farmers and institutional actors (i) how they perceive the current state and benefits of the landscape, (ii) how they foresee the future landscape under a business-as-usual (BAU) scenario, (iii) what challenges they see under the current landscape dynamics, (iv) how they want their landscapes to be in 30-years’ time,^2^ and (v) what actions they consider necessary to achieve the desired landscapes. A spatial perspective has been added to visualize the desired future landscapes—both in terms of composition (land-cover types present in the landscape) and in terms of configuration (spatial arrangement of land-cover types in the landscape). The latter is often overlooked in studies that analyze landscape dynamics based on participatory mapping or scenario building (e.g., Fagerholm et al. 2013; Robinson et al. 2016; Johansson and Isgren 2017; Kabaya et al. 2019). Including configuration in the analysis is, however, important as it influences the provision of ecosystem services as well as the degree of complementarity between them (Van Noordwijk et al. 2014; Lamy et al. 2016). In line with the literature, we assume that structurally complex mosaic landscapes provide more diverse and more complementary ecosystem services than homogeneous, segregated landscapes, which contributes to their resilience and the livelihoods that depend on them (Tscharntke et al. 2005; Castella et al. 2013).

After explaining the methodology employed in the study, the “Results” section presents landscape actors’ perceptions of the current, BAU, and desired future landscape structures, and actions necessary to achieve them. We thereby pay attention to differences between farmers from a cocoa- and oil-palm-dominated area, and differences between farmers and institutional actors. The “Discussion” section places the findings within the broader debate on landscape approaches, discussing the pros and cons, as well as the conditions of applying the methodology in practice, after which we conclude the paper.

## Methodology

### Participatory Spatial Scenario Building

This study employs participatory spatial scenario building, integrating forecasting, backcasting, and participatory mapping (Fig. 1) with a view to gaining insights into landscape actors’ perceptions of landscape dynamics and trajectories. This includes eliciting context-embedded sectorial knowledge from institutional actors and context-embedded community knowledge from farmers and local residents (van Ewijk and Baud 2009; Pfeffer et al. 2013). The approach engages actors with heterogeneous backgrounds and interests to systematically discuss plausible futures of complex, uncertain, and dynamic systems (Reed et al. 2013; Khan et al. 2015). Participatory scenario-building processes have been critiqued because outcomes do not automatically translate to change (Cairns et al. 2013). However, they facilitate the generation of insights for decision-making by allowing integration of diverse knowledge sources and perceptions, interrelationships of factors affecting systems, identification of trade-offs, visualization of impacts, and actors’ priorities (Reed et al. 2013; Kabaya et al. 2019).

While participatory scenario building has been applied in many fields, participatory backcasting is frequently used in place-based environmental applications (ibid.). Backcasting sets targets in the future, which are unachievable under current developments, and works backward to identify the requisite actions and events needed to realize the preferred future (Börjeson et al. 2006; Khan et al. 2015). Forecasting, on the other hand, predicts the likely future based on the currently prevailing conditions, without any interventions (Börjeson et al. 2006). Futures are envisioned through descriptions by interview reports and listing from workshops, sometimes modeled to provide a spatial perspective (Robinson et al. 2011; Haslauer et al. 2012; Kabaya et al. 2019). This study applied perception-based forecasting and spatial backcasting in six workshops with farmers and institutional actors to assess perceived plausible futures of a mixed cocoa-oil-palm landscape in Ghana.

### Landscape and Study Area

**Fig. 2:**
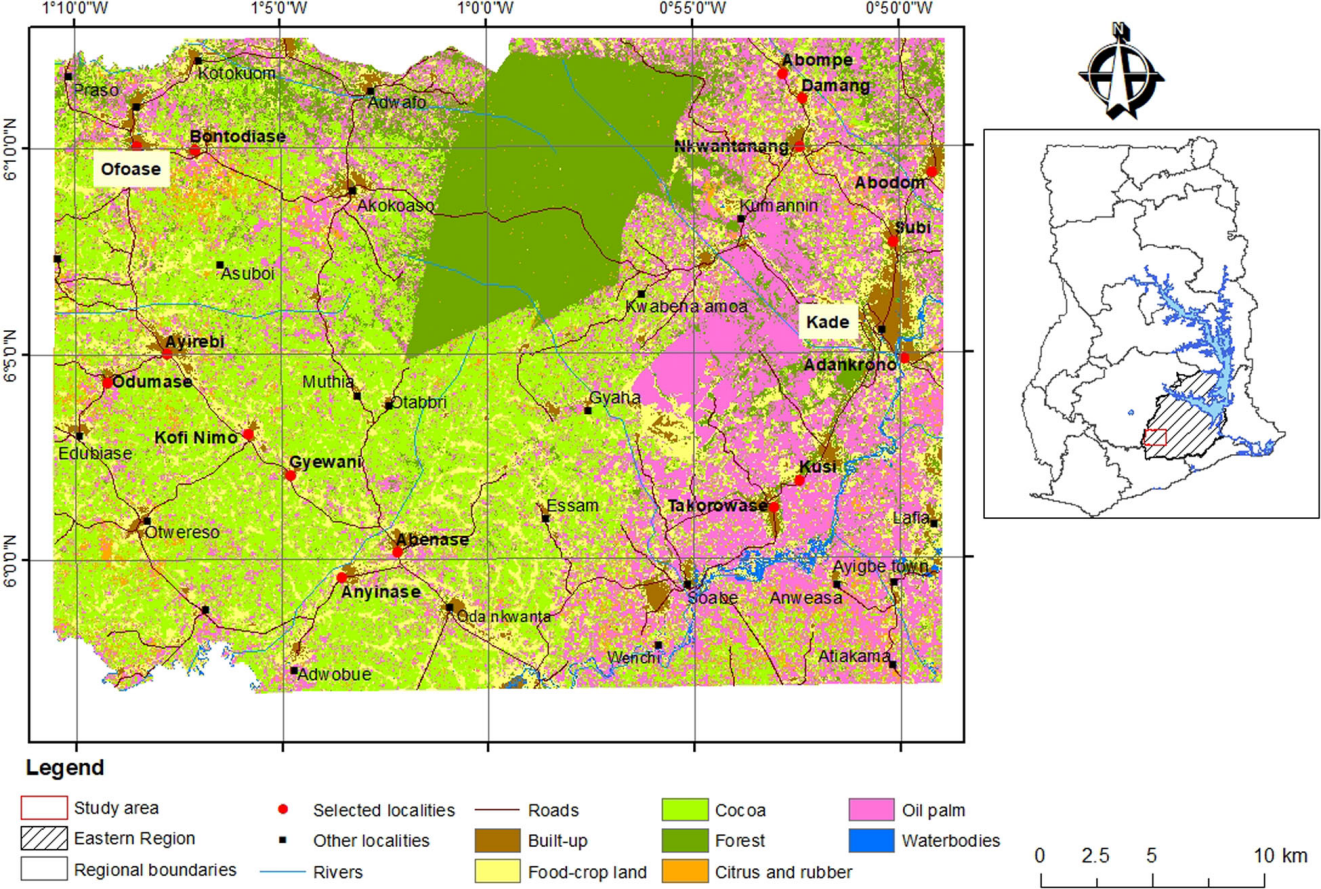
Location of the study area in Ghana showing the localities selected for the participatory spatial scenario-building workshops (Sources: Asubonteng et al. 2018, and Shapefiles—Ghana at glance, EPA)

The Akyemansa–Kwaebibirem landscape is a historically forested landscape that has undergone multiple evolutions of agricultural development with diverse combinations of food, commodity crops, and on- and off-reserve forest areas and patches. It is undergoing rapid transformation due to the expansion of cocoa and oil palm (Steel and Van Lindert 2017). Located in the southwest of Ghana’s Eastern Region (Fig. 2), it covers the area between Birim Central Municipality and Akyemansa, Denkyembour, and Kwaebibirem Districts (Asubonteng et al. 2018).

The local climate and soil conditions are conducive to cultivating tree crops—cocoa, oil palm, citrus, and rubber. The area was one of the earliest cocoa and later oil-palm frontiers in Ghana (Michel-Dounias et al. 2015). Recently, forest and tree cover has decreased substantially in size and quality, largely due to agricultural and settlement expansion (Asubonteng et al. 2018). In areas with mineral deposits, small-scale mining is also becoming a threat to agriculture and forests (ERCC 2016).

About 80% of the population in the predominantly rural landscape lives off the agricultural sector (cultivation and/or processing). Primary crops include tree crops (mainly cocoa and oil palm) and food crops (plantain, rice, cassava, and maize) (Ghana Statistical Service 2014). Cocoa is mainly for export, and oil palm and citrus are mainly targeting domestic markets, while food crops are for subsistence, with surpluses sold locally.

Most farms are not larger than 0.2–1.2 ha, although a few medium- (5–10 ha) to large-scale plantations also exist (Deans et al. 2017). From an aerial view, however, the smallholdings appear like large-scale plantations due to increasing landscape homogenization (Asubonteng et al. 2020).

### Sampling

**Fig. 3:**
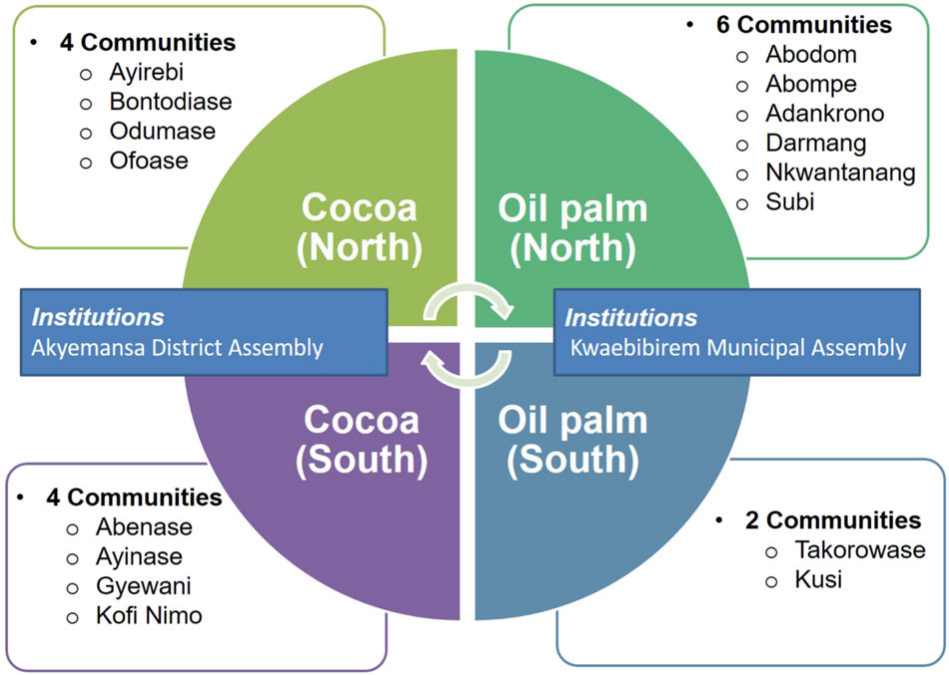
Sample design used for the selection of communities and participants

Six workshops involving two actor categories (farmers and institutional actors) were organized to capture collective actor perceptions and reflections about the current state and expected changes in their landscape (Villamor et al. 2014; Johansson and Isgren 2017). We employed a combination of stratified and random sampling approaches to ensure that participants were sourced evenly from across the area for the community-level workshops (Bryman 2012). First, we divided the landscape into a cocoa-dominated western side and oil-palm-dominated eastern side (Figs 2 and 3). Second, we divided both sides into a northern and southern part, to ensure an even distribution of selected communities. Third, five communities were randomly selected from each quadrant and invited to participate in a community-level workshop. Four such workshops were organized, each in a centrally located town, accessible by bus. For easy reference, the communities from each quadrant (Fig. 3) form a cluster, hereafter labeled after the towns hosting the workshop. These are Kade (northeast) and Takorowase (southeast), both oil-palm dominated, and Ofoase (northwest) and Abenase (southwest), both cocoa-dominated. In total, 15 communities were represented in the four workshops; one less than intended because one of the invited chief farmers was from a community outside of the sampled communities.

In consultation with the communities’ assembly persons, three people were selected from each community to participate in the scenario-building workshops. Selection was based on their knowledge of the landscape, gender, involvement in small- to medium-scale tree-crop and food-crop farming, and/or being an oil-palm processor.

Institutional actors were selected based on nominations from the district-level organizations to which they were affiliated. Of the four local jurisdictions constituting the landscape, Akyemansa District and Kwaebibirem Municipal Assemblies were chosen for the scenario workshops with institutional actors because both occupied the largest share of the predefined landscape and host the district capitals (Fig. 2). Table 1 specifies the institutions represented at the scenario workshops. Key private companies accepted invitations to participate in the workshops, but did not show up. In total, 56 farmers^3^ and 13 institutional actors participated in the scenario-building workshops (Table 1).

**Table 1:** Community clusters for scenario-building workshops and number of participants

Clusters	Number of participants
Communities^a^	
Ofoase	12
Abenase	14
Takorowase	12
Kade	18
Institutional actors^b^	
Akyemansa District	6
Kwaebibirem Municipal	7

^a^All in the Eastern Region

^b^Including the Akyemansa District and Kwaebibirem Municipal Assemblies represented by spatial planning officers and the coordinating director, the District Agricultural Development Units of the Ministry of Food and Agriculture (MoFA), the Forestry Services Division (FSD) of the Forestry Commission of Ghana (FC), the Ghana Cocoa Board (COCOBOD), and the Oil Palm Research Institute (OPRI) of the Council for Scientific and Industrial Research (CSRI)

### Participatory Spatial Scenario-building Workshops

The participatory spatial scenario-building workshops were held in July–August 2018 and lasted ~3.5 h each, following a protocol (Supplementary Material 1). The workshop protocol included an introduction, discussions of perceptions of the current state, and benefits of the landscape (step 1 in Fig. 1), and discussions of future landscape scenarios under a BAU trajectory (step 2), associated challenges (step 3), and the trajectory toward a desired landscape (steps 4 and 5), respectively. The “BAU landscape” scenario forecasts a future based on a continuation of current trends in agricultural and tree-crop policies, market systems, and cultural and societal motivations. Plenary discussions among the participants addressed composition and configuration for the current and BAU future landscapes. Composition was based on estimated proportions of each land-cover type, while configuration was ranked based on an explanation of the scale of integration and segregation ranging from 1 to 5 (see Supplementary Material 1 for details). The “desired landscape” scenarios referred to actors’ ideal vision of the state of their future landscape and were mapped. To this end, each group was given an A3 map frame of the study landscape with landmarks, including roads, rivers, and major towns, markers of different colors, sticky notepads, glue, and small paper cutouts representing the major land-cover types. Based on the maps, participants again estimated proportions per land-cover type (composition) and ranked the degree of integration–segregation (configuration). With permission of the participants, the workshop process was captured by notetaking and audio recording of plenary discussions and deliberations during the participatory mapping exercise.

After a self-introduction of the participants, workshops commenced with a brief picture-based explanation of the study objectives and clarification of landscape concepts, such as composition, configuration, and ecosystem benefits. The concepts were translated into the local language (Twi) to ensure common understanding. From there, the stepwise approach outlined in Fig. 1 was followed, an explanation of which can be found in Supplementary Material 1.

### Data Processing and Analysis

Perceived land-cover types and their estimated proportions in current, BAU, and desired future landscapes were entered into Microsoft Excel spreadsheets to produce pie charts as discussions ensued. Digital copies of participatory maps were systematically analyzed alongside the audio recordings of the group discussions. Audio recordings were translated from Twi to English and transcribed. Two members of the research team interpreted the maps independently and discussed them to ensure internal reliability (Bryman 2012). This analysis and visual comparison was based on the following themes:

• composition (number and types of land cover and proportion of landscape occupied);
• configuration (spread of land-cover types relative to geographical directions, patch numbers and distribution, and level of integration/segregation);
• geographical associations between land-cover types;
• status of the protected forest in the landscape;
• allocation of land to food crops, settlements, and small-scale and artisanal mining.

The resultant themes aligned with conventional landscape analysis informed by spatial metrics (McGarigal et al. 2012). For configuration, contiguous patches of the components of desired landscapes were counted and ranked. We assessed the relative degree of segregation by multiplying the number of land-cover types present in a map by the number of continuous patches (heterogeneity factor) and transformed the outcome to uniform values by dividing by the minimum heterogeneity factor. Landscapes with high numbers of different patches were considered integrated landscapes, while low numbers of different patches were considered segregated landscapes. Linear features like bodies (rivers) were excluded from the counts. The motivations behind the desired landscape futures and strategies to realize these futures were analyzed qualitatively from the audio transcripts and from literature.

We further ranked the participatory maps along two dimensions to analyze how differences in preferred degree of integration and segregation relate to contextual factors. These dimensions were chosen intuitively, based on the area from which the participants originated. The first dimension is the degree of rural urbanization, determined by the presence of a market town, community size, and distance from a bigger town (e.g., district capital and/or commercial center such as Kade). The second is a gradient from oil-palm dominated to cocoa-dominated area. These dimensions had no absolute values, but represent a qualitatively constructed ordinal scale. Ranking the maps along these dimensions was done based on the first author’s knowledge of the study area. This ranking resulted in four clusters of maps made by participants from:

• a rural oil-palm-dominated landscape (the rural portions of Kade and Takorowase);

• a rural cocoa-dominated area (Abenase rural);

• an urbanized oil-palm-dominated landscape (Kade township);

• an urbanized cocoa-dominated landscape (Abenase, Ayirebi,^4^ and Ofoase townships).

The desired landscapes were then analyzed by comparing the maps in the four clusters in terms of composition and configuration. Regarding the latter, specific attention was given to the number of land-cover types, cluster patterns, and adjacency of patches.

## Results

Below, we first present the results of steps 1–2 of the participatory spatial scenario-building exercise (state of the present landscape and BAU scenario, see Fig. 1) per actor group, distinguishing between farmers and institutional actors from the cocoa- and oil-palm-dominated areas, respectively. The following two steps—discussion of the challenges (step 3) and visions on the future landscape (the desired landscapes, step 4)—are presented from a comparative perspective (farmers from the cocoa-dominated area vs. those from the oil-palm-dominated area and farmers vs. institutional actors). Since the outcomes of the backcasting part (step 5) did not differ much across areas and actor groups, these are presented as a synthesis of the six workshops.

### Steps 1–2: Perceptions of Current and Future Landscapes under a BAU Scenario

#### Farmers from the cocoa-dominated area

**Fig. 4:**
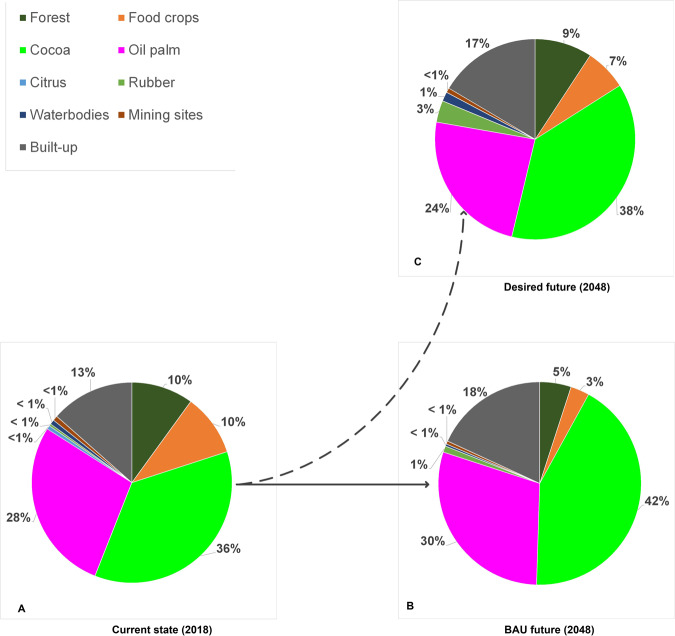
Perceived land-cover proportions by farmers in (cocoa-dominated area)

Farmers in the cocoa-dominated area perceive the landscape in 2018 to consist of cocoa (36%), oil palm (28%), built-up areas (15%), food crops (10%), forest^5^ (10%), citrus (<1%), rubber (<1%), waterbodies (<1%), and mining sites (<1%). Cocoa and oil palm—the main livelihood sources in the area—account for over half of the landscape area, with cocoa dominating (Fig. 4a).

Under BAU conditions, farmers foresee citrus to disappear due to the high vulnerability of the crop to insects, post-harvest losses, and the absence of a ready market. Like the perceived composition in 2018, farmers foresee continued domination of cocoa, oil palm, and built-up area (Fig. 4b). With an assured market, occasional government incentives, and cultural importance, they expect cocoa to further expand. Farmers in this area rank oil palm second and expect its area to slightly increase due to land suitability and frequency of yield and income. Expanding plantations of the Ghana Oil Palm Development Cooperation in Kwae and the Oil Palm Research Institute (OPRI) in Kusi, and individual farms largely account for the perceived increase in oil palm. Farmers expect built-up areas to increase due to the demand for infrastructure, accommodation for immigrant farmers and farm labor, and the shift from the traditional extended family system to a nucleus family, which requires more homes. Farmers associate a larger proportion of the built-up area with development. Compared to 2018, rubber is anticipated to marginally increase due to recent rubber plantations established by GOPDC and some farmers piloting the crop on a small scale. With a good market and secured offtake, farmers consider growing rubber trees a sound economic decision.

Farmers in the cocoa-dominated area further expect a continued decline of forest and food crops by about 50% and 70% between 2018 and 2048, respectively (Fig. 4a, b). The underlying drivers of forest decline in off-reserve agricultural areas identified by the participants include, first, the absence of direct benefits for farmers from naturally regenerated trees.^5^ Custody over these trees is in the hands of the Forestry Commission, which issues logging permits to timber operators. Both legal and illegal timber operators cause uncompensated logging damage during tree felling, causing farmers to deliberately destroy trees on farms to avoid losses. Second, forest lands are conceived as fertile sources of farming land. Hence once farms expand, forest patches will be sacrificed. Third, with growing demand for farming land, farmers sacrifice forest patches between farms in order to avoid boundary land theft and conflicts. Fourth, trees and tree clusters are thinned to ensure increased productivity of hybrid cocoa (which depends less on shade trees than conventional cocoa) and oil palm. Although considered important, farmers do no longer prioritize food-crop production due to relatively lower economic returns. In the foreseeable future, farmers expect that food-crop land will be part of the tree-crop cultivation cycle and small areas dedicated to subsistence crops in tree-crop farms and home gardens. Farmers argued that with financial resources from commodity crops, food can be purchased on the market.

Farmers in the cocoa-dominated areas expect water and mining sites to decline below 2018 levels. They attribute water area reduction to agricultural technologies that allow draining of wetlands for cocoa cultivation, siltation, and farming along waterways, which exposes the water systems to direct evaporation. Group discussions also revealed that overfertilization results in excessive weed growth in waterbodies, creating islands in them. Mining sites are expected to reduce under enforcement of the ban on illegal mining and the fact that minerals are finite resources.

#### Farmers from the oil-palm-dominated area

**Fig. 5:**
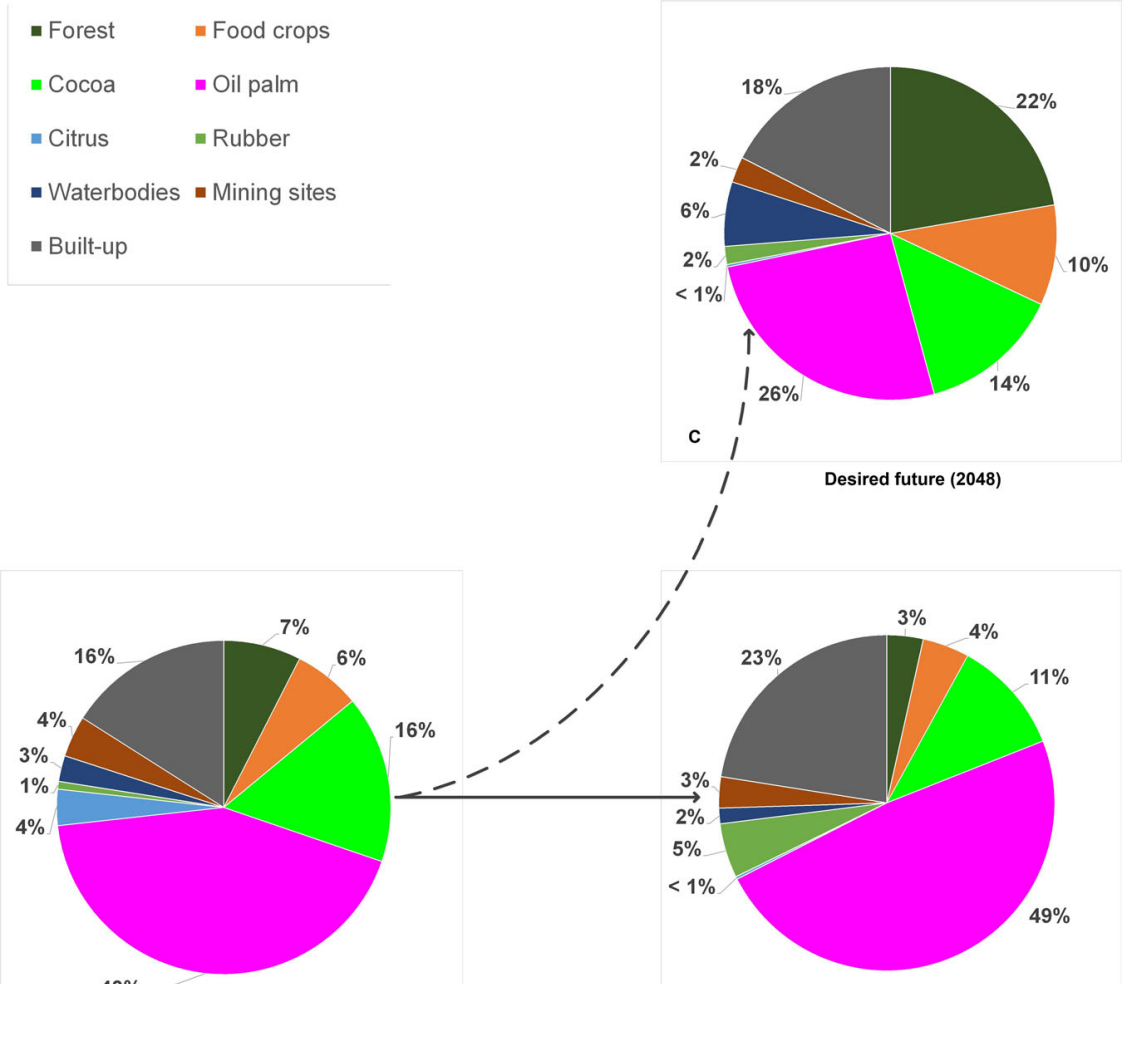
Perceived land-cover proportions by farmers (oil-palm-dominated area)

In line with the reality in which they live, farmers from the oil-palm-dominated area see oil palm as the major land cover (43%) (Fig. 5a). Built-up and cocoa-cultivated areas occupy equal shares (16%) as do forest and food crops (6–7%), but less than perceived by farmers in the cocoa-dominated area (10% each). Contrary to farmers from the cocoa-dominated area, farmers from the oil-palm side perceive sizable areas under mining (4%), citrus (4%), water (3%), and rubber (1%) (Fig. 5a).

Under a BAU scenario, farmers foresee similar land-cover types as in 2018, with oil palm alone likely to expand to almost half (49%) of the landscape, and built-up area covering 23% (Fig. 5b). The spike in oil-palm expansion is attributed to the area being ecologically more suitable for oil palm and generating higher returns than cocoa. Moreover, GOPDC and other companies are expanding their oil-palm plantations. Hence, the oil-palm-side farmers foresee a 5% reduction in lands for cocoa in the future, contrasting predictions of farmers in the cocoa area. Rubber is also expected to increase. Like farmers in the cocoa area, farmers expect a decline in mining and water areas. The latter is attributed to silt deposits from farming close to river sources. Forest and food-crop areas are also forecasted to decline, akin to the account of cocoa-side farmers.

#### Institutional actors from the cocoa-dominated area

**Fig. 6:**
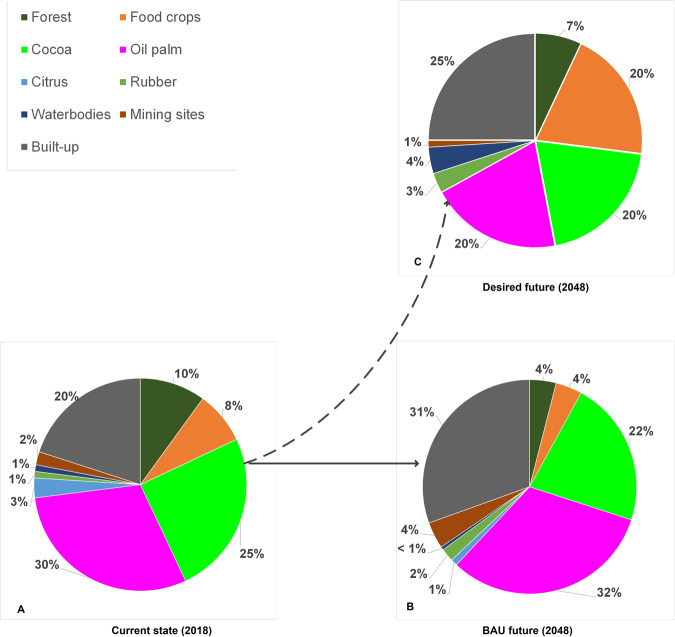
Perceived land-cover proportions by institutional actors (cocoa-dominated area)

Institutional actors in the cocoa-dominated area perceived a similar current landscape composition (2018) as the two farmer groups, with tree crops dominating more than half of the area (Fig. 6a). Yet, they differ in perceiving oil palm dominating over cocoa in the cocoa-dominated area and forests (10%) slightly dominating over food crops (8%). More than farmers, institutional actors in this area perceive larger areas under marginal land-cover types, such as citrus, rubber, mining, and waterbodies.

Institutional actors in the cocoa-dominated area foresee the future landscape under a BAU scenario to be increasingly dominated by oil palm and built-up areas (Fig. 6b). Evidenced by oversubscription to the District Assembly’s oil palm seedling supply programs, they perceive a renewed interest in the cultivation of oil palm, which they attribute to relatively high financial returns and the opportunity to harvest every 2 weeks. They therefore expect that some cocoa fields will be taken over by oil palm. They further expect increased built-up area due to population increase and growing demand for infrastructure. Settlement expansion is expected to absorb the adjoining cocoa fields and food-crop areas, leading to a reduction of both below 2018 levels. Moreover, they foresee 60% forest loss notably in marshy areas and at the forest reserve margins. Similar to farmers’ forecast, they foresee a decline in food-crop areas because people are reserving fewer and smaller plots for food production areas in their farm than before and rely on food imports from neighboring areas (Fig. 6b). Institutional actors in the cocoa-dominated area further foresee expanding mining and rubber areas, while waterbodies and citrus will decrease to negligible proportions.

#### Institutional actors from the oil-palm dominated area

**Fig. 7:**
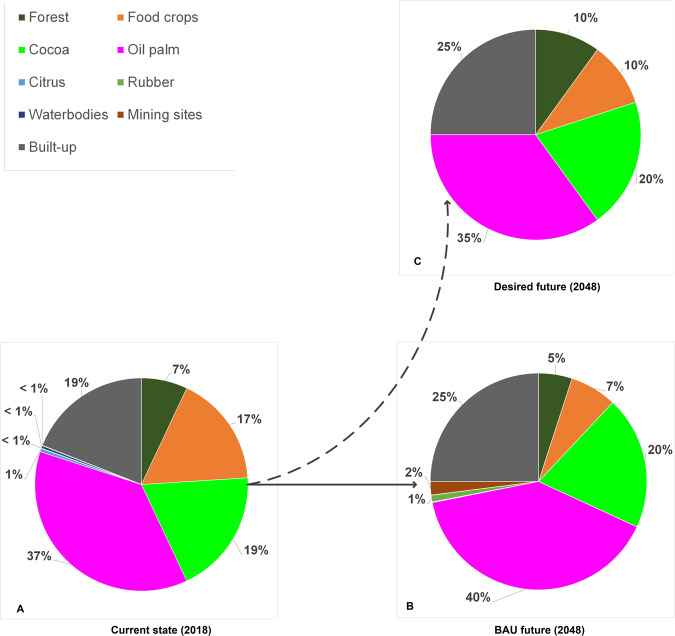
Perceived land-cover proportions by institutional actors (oil-palm-dominated area)

Like their counterparts from the cocoa side and the farmers in their area, institutional actors at the oil-palm side perceive the current landscape as being tree-crop dominated, with oil palm covering 37% (Fig. 7a). They estimate food-crop areas at about 17% of the landscape, more than any other actor. In their view, forests occupy a mere 7% of the landscape, similar to the estimates of farmers in the cocoa area (Fig. 4a). The other land-cover types are regarded as negligible, covering areas of less than 1% of total each.

These institutional actors foresee a BAU future without citrus and increased tree-crop predominance (Fig. 7b). They forecast oil palm and cocoa to expand due to private sector and government interventions. They expect cocoa expansion to be driven by programs of the Ghana Cocoa Board (COCOBOD) that incentivize farmers through assured markets, free replanting of old cocoa fields, pest control, and other measures. However, oil-palm-side institutional actors also believe that high and frequent incomes from oil palm, coupled with GOPDC’s upscaling of its outgrower scheme, OPRI’s development of high-yielding oil-palm seedlings, and the growing number of processing companies will encourage the youth to engage in oil-palm farming. With increasing immigration and population growth, the institutional actors expect built-up areas to increase proportionally. Identical to forecasts by other actors, they anticipate that mining areas will expand, despite the nationwide ban on small-scale mining. They also expect rubber areas to increase due to new plantations by GOPDC and piloting farmers. In line with other actors, they foresee that these expansions will result in less land under food crops and forest (Fig. 7b). Institutional actors predict that riparian deforestation will increase due to mining. They expect waterbodies to decrease due to dry-season farming along waterways, which results in increased evaporation, siltation, and eventual drying up.

#### Perceived landscape configuration under current and BAU scenarios

**Fig. 8:**
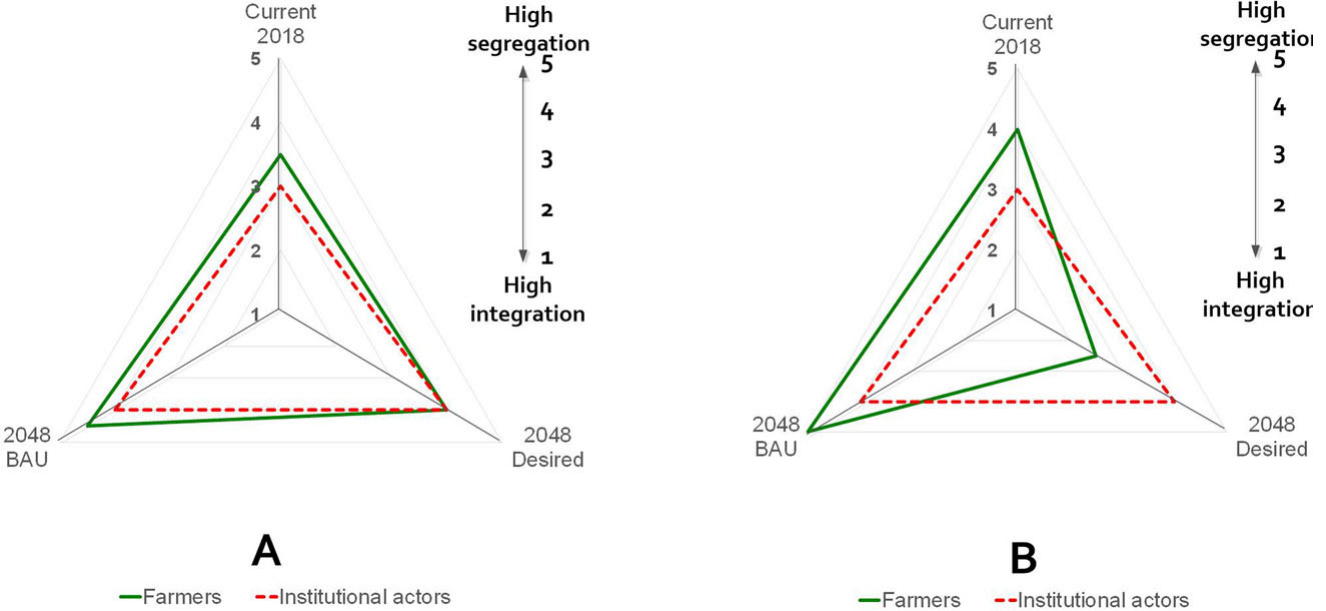
Actors’ perceptions of levels of integration/segregation in current (2018), BAU (2048), and desired (2048) future landscapes in cocoa- (**a**) and oil-palm- (**b**) dominated areas

Farmers and institutional actors differ in their perceptions of landscape integration/segregation. Farmers in the cocoa-dominated area positioned the landscape at 3.5 on the integration–segregation continuum, slightly more segregated than institutional actors’ score 3 (red lines) (Fig. 8a). Farmers in oil-palm areas, or areas close to those (e.g., Abenase in the cocoa-dominated area) tend to position the landscape at early stages of segregation (score 4) (Fig. 8b).

In 30-years’ time, however, all actors foresee more segregation (3.5–4.5) under a BAU scenario. Farmers in the cocoa-dominated area perceive a level of 4 on the integration–segregation continuum, suggesting modest segregation. Farmers from the oil-palm-dominated area and the surroundings (Kade and Takorowase) forecast extreme segregation (level 5) under a BAU future. As will be seen later, for farmers and institutional actors in the cocoa area, the degree of segregation does not differ much from their desired landscapes, but farmers from the oil-palm-dominated areas tend to prefer a more integrated landscape (score 2) than they expect will evolve under a BAU scenario (score 4).

#### Benefits from the landscape

Farmers perceived a variety of benefits from the landscape, and these perceptions were largely similar across the cocoa- and oil-palm-dominated side of the study area (Table 2). They focused mainly on provisioning services, with farmers stressing their subsistence and commercial value, and institutional actors emphasizing income and employment. Farmers explicitly mentioned mineral (gold and diamond) as benefits, which are usually not included in accounts of provisioning services; the institutional actors did not mention these at all. Only a few supporting and regulating benefits were mentioned, and by farmers only, confirming that institutional actors are more alienated from the landscapes in which they work. Cultural benefits were not mentioned at all.

**Table 2:** Actors’ perceived benefits from the landscape, categorized according to The Economics of Ecosystems and Biodiversity (TEEB) classification of ecosystem services (http://www.teebweb.org/resources/ecosystem-services/)

	Cocoa-dominated		Oil-palm dominated	
	Farmers	Institutional actors	Farmers	Institutional actors
Provisioning services				
Food	Food crops, snails, mushrooms	Food crops	Food crops, fruits, meat (including game), snail, crab and mushrooms	Food crops, snails and mushrooms
Raw materials	Cocoa, oil palm, citrus, rubber, mineral deposits	Cocoa, oil palm, citrus, rubber, diamond, sand	Timber, cocoa, oil palm, citrus, rubber, gold, diamond	Cocoa, oil palm, citrus, rubber
Medicinal resources	Medicinal herbs		Medicinal herbs	
Fresh water			River, streams and marshy lands	
Contribution to wellbeing	Income	Income	Income	Income
				Employment
Regulating services				
Local climate and air quality			Fresh air	
Moderation of extreme events			Windbreaks	
Pollination	Pollination			
Erosion prevention & maintenance of soil fertility	Soil fertility			
Biological control	Pest control			
Supporting services				
Habitat for species			Land for forests and trees	
Maintenance of genetic diversity	Biodiversity marshy areas			

### Step 3: Perceived Challenges Due to Landscape Change

Farmers and institutional actors perceive several environmental threats associated with the current state of the landscape, including significant land degradation and declining water quality and quantity due to mining and shifts in season, and declining availability of non-timber forest products due to deforestation (Table 3). All farmers lamented about low soil fertility, drying up of waterbodies, and local increase in alien species hitherto absent in the landscape. Aside from the shared perceived challenges, there were some context-specific threats. For example, oil-palm-dominated areas are faced with increased prevalence of pests and diseases, increased bushfires, and wood scarcity, while declining pollinators were mentioned only in the cocoa-dominated areas.

**Table 3:** Actors’ perceived challenges in the current state of the landscape (F = farmers, I = institutional actors)

Threats	Cocoa-dominated area	Oil-palm dominated area
Environmental threats		
Decline in fruits, wildlife, snails and mushrooms	F, I	F, I
Polluted streams and rivers	F, I	F, I
Climate change (shift in seasons and heavy rains)	F, I	F, I
Land degradation (mining)	F, I	F, I
Dwindling water resources	F	F
Local invasion of alien snails and grass species	F	F
Low fertility	F	F
High disease and pest presence		F
Increased bushfires		F
Scarcity of timber (wood)		F
Declined pollinators	F	
Socio-economic threats		
High food cost	F, I	F
Food shortage	F, I	F
Low yield per acre	F, I	F
Scarce and pricy land (increasing population)	I	F, I
Property destruction (strong winds)	F	F
High cost of farm inputs	F	F
Low price in the bumper season		F
Off-season poverty		F
Increased labor cost (due to ASM and high labor demand)		F
Plastic waste infested lands	F	

Similar to environmental threats, institutional actors perceived less socioeconomic threats than farmers. High food cost, limited availability of locally produced food, low yields per hectare, and land scarcity were perceived by both, but the list of socioeconomic threats experienced by farmers was much longer, especially in the oil-palm-dominated area (Table 3).

### Step 4: Spatializing Actors’ Desired Future

#### Farmers’ desired future landscapes

**Fig. 9:**
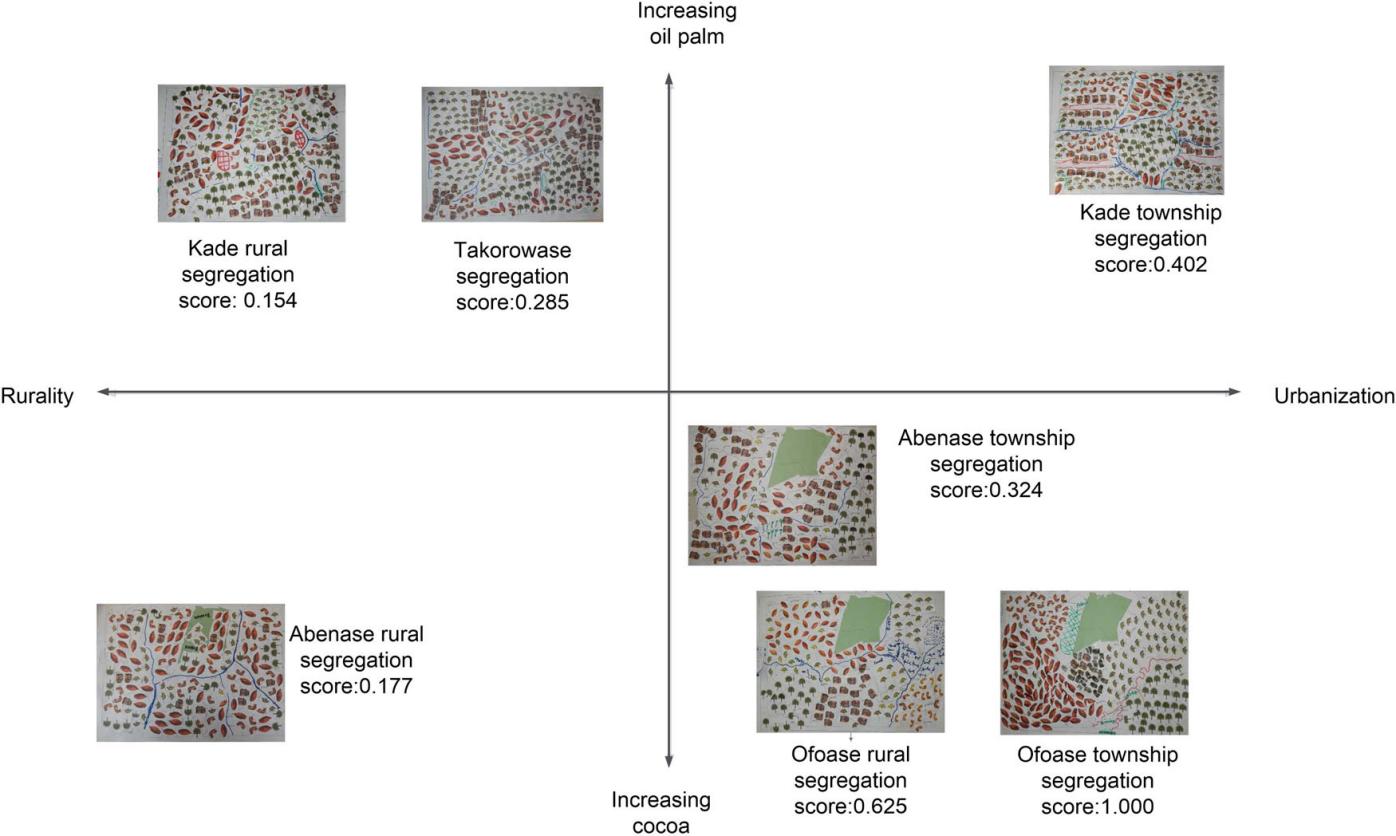
Farmers’ preferred degree of integration/segregation and locality characteristics (degree of urbanization/tree-crop dominance) (see supplementary material 2 for the maps in more detail)

The participatory maps from the spatial scenario-building workshops provide insights into both composition and configuration of farmers’ future landscapes. As the participatory maps show, forest, cocoa, oil palm, food crops, built-up areas, and waterbodies are common features in farmers’ desired landscapes (Fig. 9; Supplementary Material 2). However, the preferred spatial distribution of land-cover types varied among actors from different landscape types, with “dominant tree crop in respondents’ area of residence” and “proximity to bigger towns with markets” being the main explanatory factors.

Farmers in the cocoa-dominated area prefer a landscape devoid of citrus and dominated by the major tree crops (cocoa and oil palm) and built-up areas (Fig. 4c). In their desired landscape, cocoa, built-up areas, and rubber are larger than the 2018 levels, but lower than under the BAU scenario, while oil-palm areas will be below the 2018 sizes. Farmers desire a reduction in forest and food-crop areas relative to 2018, but less than expected under the BAU scenario. In their desired landscape, the area allocated to mining remains equal, so that it will not claim more arable lands and riverbanks than in 2018.

Cognizant of the environmental, economic, and social risks associated with the BAU scenario, farmers in the oil-palm-dominated area aspire a different desired landscape. They wish significantly less land to be allocated to oil palm (26%), while increasing the land allocated to forest (22%) and food crops (10%) (Fig. 5c). They hope that a larger forest area contributes to the restoration of biodiversity and associated ecosystem services that are lacking in the current landscape. Farmers expect that only marginal increases in food-crop areas are needed because production can be augmented with fertilizer, improved management practice, and technology. Moreover, food deficits are already being filled with imports from neighboring food-growing areas. The desired proportion of cocoa (14%) is lower than that of the 2018 area, but larger than forecasted in a BAU scenario (Fig. 5b, c). The relative proportions of areas envisaged for cocoa and oil-palm production indicate that cocoa is not a priority for farmers in the oil-palm area. Built-up area is preferred to increase relative to that of 2018, but not as much as under the BAU situation. This reflects the need to provide adequate space for accommodation and services to the increasing population, and corresponds with the desired future landscape in the cocoa area. Farmers in the oil-palm-dominated area aspire to have fewer mining and citrus areas compared to 2018. While acknowledging the livelihood opportunities and contribution offered by mining, there are great concerns about water pollution and productive land degradation. Citrus is less desired due to a lack of markets and persistent pest and disease challenges. The few citrus areas likely to be left will be owned by juice-manufacturing factories and farmers who have dealings with the company. The proportions of water and rubber in the desired landscape double relative to their estimated occurrence in 2018. This can be explained respectively by the fact that oil palm yields more in wetlands and close to water sources, and that rubber is expected to generate income.

In terms of configuration, the maps reveal six remarkable patterns, similarities, and differences. First, farmers from more distant rural areas prefer a more integrated landscape (average segregation of 0.204) compared to those from more urbanized landscapes (0.518). Second, the preferred degree of segregation increases with growing cocoa dominance (average segregation score of 0.531) among cocoa-side farmers compared to preferences of farmers from oil-palm areas (0.280) (Fig. 9). The latter’s preference for more integrated landscapes is due to having experienced the consequences of increasing landscape homogenization. Third, the maps reveal a preference for increasing forest cover with growing urbanization. Fourth, all participating actor groups prefer land for food crops close to the settlement areas, irrespective of urbanization degree or dominant tree crop. However, farmers from rural areas prefer land for food crops to be more integrated with other land-cover types, while those from urbanized areas allocate land for food crops segregated from other land uses. Fifth, only participants from rural oil-palm areas and urbanized cocoa landscapes assign land to artisanal and small-scale mining in their desired landscapes, and all do so in concentrated patches along waterbodies. Sixth, the forest reserve remains protected in the desired landscapes of those in cocoa-dominated areas, but faces slight-to-heavy encroachment in the desired landscapes in rural areas (both cocoa- and oil-palm dominated). The desired landscape of farmers near Kade township depicts total conversion of forest reserve to cocoa, but value attached to tree cover near settlements (see below).

A more detailed analysis of cluster-specific features in the desired landscapes can be found in Supplementary Material 3.

#### Institutional actors’ desired future landscapes

All institutional actors prefer a tree-crop-dominated landscape (but without citrus due to high losses and a lack of farmers’ interest and domestic demand) and food crops near settlements (Figs 6–7c). At both the cocoa- and oil-palm-dominated sides of the landscape, institutional actors want both oil palm and cocoa, whereby those from the cocoa-dominated Akyemansa District refer to the frequent income from oil-palm harvests throughout the year. Institutional actors at the cocoa side are more concerned about future food security than those at the oil-palm side and assign a larger area to food crops (20%) than those at the oil-palm side (10%). The latter argue that food needs can be met with food from the Kade market and tree-crop establishment cycles during which food crops are interplanted with tree crops in the early years of establishment. In both areas, the forest reserve remains fairly intact, but in the desired future of the cocoa-dominated Akyemansa District, the northern portion of the forest reserve is sacrificed to pilot rubber trees, in expectation of new economic opportunities. Instead, new forest patches are created in the off-reserve area to meet the need for timber and other provisioning services. Mining (1%) is only found along a river in the southwestern corner of institutional actors’ desired landscape in Akyemansa District, but no longer in Kwaebibirem District out of fear of water pollution and farmland degradation. Whereas settlements and built-up area cover similar areas in the desired landscapes of institutional actors at both sides of the landscape (25%)—the increase of which they consider inevitable due to population growth and infrastructural needs for economic development—they are clustered throughout the desired landscape in the cocoa-dominated area and scattered dwellings in the desired future of institutional actors in the oil-palm-dominated area. Institutional actors at the cocoa side prefer water to increase to 4% for its relevance to agriculture and human well-being. Those from the oil-palm side assume water to be an integral part of forest and hence did not map it separately.

In terms of configuration, institutional actors from both areas prefer landscapes in the middle range of the integration–segregation continuum, differing from farmers in the same areas. The segregation score of 0.238 in the cocoa-dominated areas is much lower than the average score among farmers (0.531), while the score of 0.31 in the oil-palm-dominated area is slightly higher than the average score among farmers (0.280). Whereas the degree of segregation is evenly spread over the oil-palm-dominated Kwaebibirem District, the desired future of the cocoa-dominated Akyemansa District reveals a more complicated pattern. The eastern side of the desired landscape in Akyemansa, where oil palm and clustered settlements dominate, is much more segregated than the mosaic landscape in the west, where cocoa, food crops, forests, and a bit of oil-palm alternate.

### Step 5: Walking the Talk: How to Achieve the Desired Landscapes (Backcasting)

“*Achieving desired futures is not a one-time project, but one that involves several phases with feedback loops. It requires the inclusion multiple landscape actors*” (Farmers from Kade in Akyemansa–Kwaebibirem landscape)

This section presents landscape actors’ views on how to achieve the desired future landscapes, based on the aggregated outcomes of the six workshops. All participants realize that transforming the landscape to the desired futures is a process involving several phases with feedback loops and the inclusion of multiple landscape actors.

#### Stage 1: sensitization

Participants believed that participatory spatial scenario building should be applied more broadly to enhance a shared understanding of landscape dynamics and concerns, and to achieve stakeholder buy-in for changing detrimental practices. They felt that discussing the future of the landscape under a BAU scenario made stakeholders aware of the consequences of their actions (e.g., clear felling and excessive use of agrochemicals). Creating awareness should be done at different levels and with different stakeholder groups, such as youth in schools, farmers, private sector, traditional authorities, and civil servants. Participants suggested that such processes should be steered by researchers and academics with the backing of governing authorities in the landscape, including traditional authorities.

#### Stage 2: actor engagement in planning

Achieving the desired future landscapes requires an inclusive planning process in which the maps are presented to representatives of various stakeholder groups for discussions on trade-offs, compromises, and compensation. Agreed outcomes of these meetings should result in amended maps that should be resubmitted for verification and inputs in town hall meetings with a broader range of stakeholders. Key to the successful implementation of the process is the inclusion and leadership of traditional authorities as the owners of the lands; of assembly persons as bridging actors between landscape dwellers, traditional authorities and local government; and of the District Assembly representing statutory government. This process should be devoid of the usual mistrust between government and local chiefs. The resultant landscape vision should be translated into proper land-use and development plans, which should be documented at the District Assembly and made available publicly with assembly persons designated as intermediaries.

#### Stage 3: designing policies and laws

This stage comprises policy formulation based on actors’ agreed desired future landscape. The policy should be backed by laws and regulations that spell out rights and responsibilities and sanctions for noncompliance, and reflect the traditions or customs that govern landscape resources such as forest and water. The policies and supporting laws should be made publicly available via the National Commission for Civic Education in a joint education effort with churches, schools, and traditional leaders. Existing agricultural policies that prioritize cocoa and oil palm over food crops should be revised to encourage the production of food crops. Forest-based policies should respect farmers’ rights to timber trees on their farms. Policy continuity beyond electoral cycles was considered essential. Considering the landscape-wide changes proposed in the plans, once implemented, they should be carried out until the end to prevent further landscape degradation. Law enforcement was considered crucial to achieving the desired landscapes, with sanctions applied, irrespective of the person who breaks the law. Actors, however, believe that laws will be respected if developed in a bottom-up manner.

#### Stage 4: implementation

The actual implementation of the landscape plans would include the establishment of a joint interinstitutional coordinating board with members from traditional authorities, community representatives, the Forestry Commission, agricultural departments, and the security force (police and judiciary). The mandate of this team will be to supervise land demarcation and land-use allocations. Provision of tree seedlings for forest restoration, enforcing bans on mining and chainsaw milling, and reclamation of mining sites are considered critical. Farmers were more explicit than institutional actors about addressing issues of compensation of farmers in the case of relocation or changing land use.

#### Stage 5: monitoring, evaluation, and adjustments

Each stage should be monitored for progress and feedback loops, resulting in adaptation of the plans and implementation process where needed.

#### Barriers

Landscape actors recognized the following possible obstructions to realizing their desired landscapes:

- Implementation costs associated with process logistics and compensation of farmers and others who need to move to other locations.
- A lack of political will to devolve authority to landscape dwellers and a lack of continuity, particularly after a change of government.
- Continued population growth due to immigration and uncontrolled child births, resulting in a greater demand for land for production and settlement.
- Overlapping land tenure systems potentially resulting in confusion and conflict when implementing a landscape approach. This already happens when chiefs allocate land to uses other than those planned by the local government authorities.
- Nonenforcement of laws that protect natural resources and guide resource use. Both statutory and traditional authorities frequently interfere when sanctions for breaches are imposed, leading to impunity and resource degradation.
- The focus on commodity crops as the main economic driver in landscape. The assured markets and prioritization of commodity crops by all actors in the landscape jeopardizes other landscape components.

## Discussion and Conclusion

This study aimed to present and apply a participatory spatial scenario-building methodology to uncover landscape actors’ views of landscape dynamics and desired landscapes. Below, we first discuss the findings on perceived composition and configuration and how these reveal farmers’ preference regarding sparing (segregated landscapes) or sharing (integrated landscapes). Next, we reflect on the pros and the cons of the methodology.

### Perceived Composition and Configuration

By and large, actors’ perceptions of the composition of the current landscape coincide with the satellite image classification of the same area by Asubonteng et al. (2018)—an area dominated by the tree crops cocoa and oil palm, where tree crops and built-up area are increasing, and forest and areas for food crops are declining. However, all actors, except the farmers in the cocoa-dominated area, tend to overestimate the dominance of oil palm in the current landscape. This contrasts with the 2015 land-cover map by Asubonteng et al. (2018) based on remote sensing, which shows that cocoa prevails. The observed discrepancy can be attributed, first, to the observed trend that the oil-palm area is rapidly increasing. Second, actors’ perceptions may be biased toward experiences in their immediate environment (e.g., oil-palm dominated) and the lack of a bird’s eye view to holistically assess the entire landscape. This is also related to oil palm often being planted as monoculture, whereas cocoa is generally interspersed with food crops, tree clusters, and shade trees, and as such does not create the impression that it dominates the landscape.

Rural urbanization is often overlooked in both the literature on land sharing and sparing (Perfecto and Vandermeer 2010; Phalan et al. 2011; Tscharntke et al. 2012) and ILA literature (Bürgi et al. 2017). All actors expect the built-up area to increase, reflecting a process of increasing rural economic growth, infrastructural development, and rural urbanization as foreseen in Ghana’s national decentralization program (Owusu 2005).

In terms of configuration, institutional actors from both cocoa- and oil-palm-dominated areas perceive the current landscape to be in the middle of the integration–segregation continuum, whereas farmers generally indicate higher levels of segregation. Farmers’ perceptions correspond with the findings of Asubonteng et al. (2020), who positioned the landscape on the segregation side of the integration–segregation continuum. We attribute the different perceptions to institutional actors being less familiar with the landscape than farmers because they often originate from other areas and pursue an urban-based livelihood.

The desired landscapes were context-specific, depending on actors (farmers vs. institutional actors), dominant tree crop in the landscape where participants live (cocoa vs. oil palm), and degree of rural urbanization. For composition, we observed differences between farmers and institutional actors, which presumably relate to greater familiarity through interaction and experiential knowledge of the landscape among farmers (Natori and Chenoweth 2008). Similar contrasting views between farmers and institutional actors were found in a disaster management study in northern Ghana (Kusakari et al. 2014).

The results show that farmers from the cocoa area prefer a more segregated landscape (land sparing), whereas oil-palm-side farmers prefer a more integrated landscape (land sharing). Farmers’ preference for segregation in cocoa areas is motivated by crop damage caused by timber operators (Marfo and Schanz 2009; Ros-Tonen and Derkyi 2018), conflicts over unfarmed areas (Derkyi et al. 2014), and prospects of greater efficiency and higher yields (income) (Phalan et al. 2011; Cannon et al. 2019). Contrastingly, farmers in the oil-palm area have experienced the negative consequences of a highly segregated landscape, despite the higher incomes that tree crops generate. Such trade-offs include declining food production, increasing food cost, and declined availability of ecosystem services and access to timber and non-timber forest products (Pfund et al. 2011; Egan and Mortensen 2012; Tscharntke et al. 2012; Castella et al. 2013; Anderman et al. 2014). Different perspectives, such as those between farmers from the cocoa- and oil-palm-dominated areas, affect the direction and outcome of multistakeholder negotiations as envisaged in ILAs. This shows that there is no one-size-fits-all solution; even within a single landscape, preferred directions are context-specific.

### Methodological Considerations

The applied methodology revealed several merits of participatory mapping and scenario building in relation to landscape and development planning that have also been documented elsewhere (e.g., Boedhihartono 2012; Pfeffer et al. 2013; Villamor et al. 2014). It proved to be useful, first, to make actors aware of landscape issues and long-term implications of current trends in the landscape. Spatializing the future made actors aware of the competition for space and trade-offs between competing land uses. Mapping and scenario building encouraged farmers to look beyond their farm and to think on the longer term. This helped them to visualize how much land they allocate to a particular land use, and how this will be distributed and arranged in the landscape. It made actors cognizant of the need to address challenges in the landscape and to achieve consensus on land-use allocation. Second, the methodology made farmers’ collective knowledge and different perspectives explicit, both in narratives and maps. The collective nature of this process results in validated and negotiated knowledge, realistic rather than idealistic discussions, and a spatially explicit outcome of the process. Third, the methodology includes farmers in deliberations on landscape governance and visions for the future, thus enhancing their negotiation power.

These advantages make the methodology a promising entry point for the implementation of ILAs. Indeed, the stepwise approach proposed by workshop participants to address landscape concerns resembles ideas about ILAs and the design principles developed by Sayer et al. (2013) (Supplementary Material 4). However, a notable difference is the key role assigned to statutory government actors and traditional authorities. In this respect, participants’ proposal aligns more with a jurisdictional approach, which is a form of integrated landscape governance in which “the landscape is defined by policy-relevant boundaries and the underlying strategy is designed to achieve a high level of governmental involvement” (Stickler et al. 2018). This makes sense in Ghana’s context, which is characterized by multilayered and hierarchical governance (Derkyi 2012) in which landscape issues cannot be discussed without involving traditional authorities and government actors, such as the Forestry Commission, District Assemblies, and the district departments of Food and Agriculture (Foli et al. 2018; Ros-Tonen and Derkyi 2018). Another difference between the approach proposed by the participants and the literature on ILAs is that the former is more explicit about landscape negotiation as a process with a time path that distinguishes between short-, medium-, and long-term activities and outcomes.

The key to successful application of the methodology is the room for exploring what works in a participatory spatial scenario planning process engaging different actors. Although the backcasting approach (Fig. 1) was piloted before without a spatial component in the learning platforms of the Inclusive Value Chain Collaboration project (inclusiveVCC.wordpress.com), inclusion of the spatial component required some experimentation and ad hoc decisions and adaptations in the field. Practitioners should be cognizant of context-specific realities during actor engagement workshops. First, language can pose a challenge as complex concepts like landscape, configuration, and composition had no direct equivalents in the local language, Twi. For example, the term “landscape”, a key concept in the approach, lacks a universal understanding in terms of scale and extent, even among scientists (Sayer et al. 2013). Using the scaled integration–segregation continuum (Supplementary Material 1) facilitated communication about actors’ perceptions of configuration to which they are not familiar. Second, it had to be made clear to farmers at what scale the landscape should be understood, particularly in discussions about configurations (Karner et al. 2019). Without such common understanding, a farm with mixed crops may be misconstrued as an integrated landscape, whereas that same farm is only a small landscape component at a higher-scale level. Third, the smooth facilitation of such multiactor processes largely depends on the facilitator’s skills and knowledge about the issues at stake in the local context. Such skills and knowledge enable facilitators to ask probing questions, to earn actors’ confidence in the processes, and to build trust. Knowledge of the local language is a precondition in this respect. Fourth, the selection of participants was to some extent gender inclusive, but not gender specific; ideally both male and female participants should be selected from each social group and mapping done in separate groups for better insights into gender differences regarding the desired landscapes.

Despite the promises of the approach, some methodological challenges are worth a reflection. First, the values of the segregation scores calculated from the participatory and qualitative maps of desired landscapes are a proxy that can be used for ranking but not in absolute terms. Using gridded map frames with cell sizes equivalent to those of the symbols could enhance accuracy in future estimates. Second, we determined the level of rural urbanization based on the place of residence of the participants, although they were not always from the same locality. We assumed that those coming from elsewhere would adapt to the majority view, while this might not always be the case. However, this did not compromise the validity and reliability of the results, which were consistent across participants and workshops from similar landscapes. Third, the participatory spatial scenario-building process can be time-consuming, and spreading over multiple sessions is recommended. This may avoid fatigue and loss of interest among participants. However, multiple sessions will have cost implications, hence the need to seek a balance between time, number of participants, and cost. Finally, expectation management is key in such processes, in order to avoid disappointment among participants if actions to achieve their desired landscapes are not implemented. Facilitators should be cautious not to make false promises, which could compromise actors’ willingness to engage in participatory processes in the future.

In conclusion, the participatory spatial scenario-building approach successfully helped facilitate multiactor dialogs in landscapes about landscape dynamics, trade-offs, desired futures, and actions deemed necessary to achieving them. As such, it lends itself as a useful approach for operationalizing ILAs and incorporating the views of landscape actors. While we do not seek to present our findings as blueprints for the future of the landscape, we aimed to make clear that local policies and global debates should recognize the diversity in actor choices and the context specificity of motivations behind these choices.

## Supplementary information

Supplementary Material

## Footnotes

1 “Mosaic landscapes”—also referred to as “working landscapes” (Kremen and Merenlender 2018) and “smallholder landscapes” (Clough et al. 2016; Grass et al. 2020)—are defined in this paper as structurally complex spaces of variable scale that accommodate different interacting land-cover types, of both natural and anthropogenic origin. In this study, the scale is defined by the area used in the participatory mapping exercise (see “Methodology” section). Because of their multifunctional nature, mosaic landscapes offer a greater variety of ecosystem services than more homogeneous landscapes such as those dominated by large-scale monoculture plantations.

2 A 30-years’ timeframe was chosen to align with satellite-based studies of landscape dynamics covering 30 years into the past (Asubonteng et al. 2018, 2020).

3 Where “farmer” is used, this includes male and female farmers and may include an oil-palm processor. The latter is commonly female.

4 Participants from Ayirebi township participated in the Ofoase township workshop.

5 Farmers are only entitled to fell and sell trees that they planted themselves.
