# Supplementary material for: Envisioning the Future of Mosaic Landscapes: Actor Perceptions in a Mixed Cocoa/Oil-Palm Area in Ghana
Source: Environ Manage. 2020 Oct 15;68(5):701–19. doi: 10.1007/s00267-020-01368-4 (PMC8560681; doi:10.1007/s00267-020-01368-4)
Supplement: Supplementary file 1 — Supplementary Material [file 267_2020_1368_MOESM1_ESM.docx]

# Supplementary material 1: Protocol for the participatory spatial scenario-building workshops

**Step 1** in the scenario-building exercise (Fig. 1 in the paper) engaged the participants in a discussion about the current state of landscape composition, configuration, and ecosystem benefits. Composing land-cover types were listed and their perceived proportion of the landscape estimated. To facilitate understanding of notions of configuration, we used a pictorial representation of the spatial integration-segregation continuum in landscapes adapted from van Noordwijk et al. (2012) scaled from 1 to 5, with 1 being the least segregated landscape and 5 the most segregated (Fig. S1).


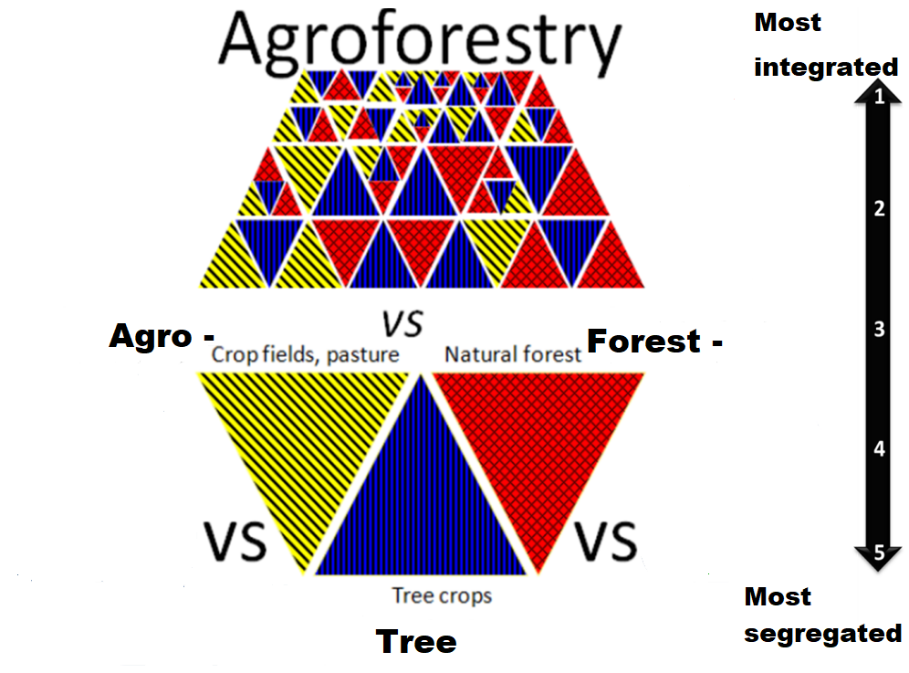


**Fig. S1** A pictorial representation of spatial integration-segregation scale used in scenario workshops (red = forest, blue = tree crops and yellow= food crop) (adapted from Van Noordwijk et al. 2012).

**Step 2-3** (Fig. 1) discussed the future of the landscape under a business-as-usual (BAU) scenario, highlighting foreseeable challenges in the landscape in 30 years’ time following current trends of market-driven smallholder engagement in tree-crop production and expansion, policies and market dynamics.

In **Step 4** (Fig. 1), participants designed their desired future landscape in terms of both composition and spatial arrangement (configuration), employing participatory spatial backcasting, bearing in mind the changes identified in the current landscape.

For participatory spatial backcasting, each group was given an A3 map frame of the study landscape with landmarks including roads, rivers and major towns; markers of different colors; sticky notepads; glue; and small paper cut-outs representing the major land-cover types. Participants were allowed to add other than the pre-produced land-cover types by using shades that should be labelled in map legends. For this exercise, participants in each workshop, except in Takorowase, were further divided into those with a majority from bigger towns and those from rural locations. Participants’ turnout in Takorowase was initial low due to a heavy rain. With the tools provided, the groups constructed the desired landscapes considering the components and their arrangements. Recognizing the difficulty for the participants to spatially construct the landscape to scale, they first collectively estimated percentages of land allocated to each land-cover type. While the participants worked on their desired landscapes, voice recorders were placed on their table to capture the discussions resulting in maps.

**Step 5** guided the subsequent plenary discussions to focus on perceived actions and timelines required to achieve the desired landscapes, barriers to achieving the desired outcome, and ways to remove them.

Participants presented and discussed their maps in a plenary, enabling comparison with outputs from other group sessions. Participants’ permission was sought before taking photos and making audio recordings.

# Supplementary material 2: Actors’ envisioned futures participatory maps

| 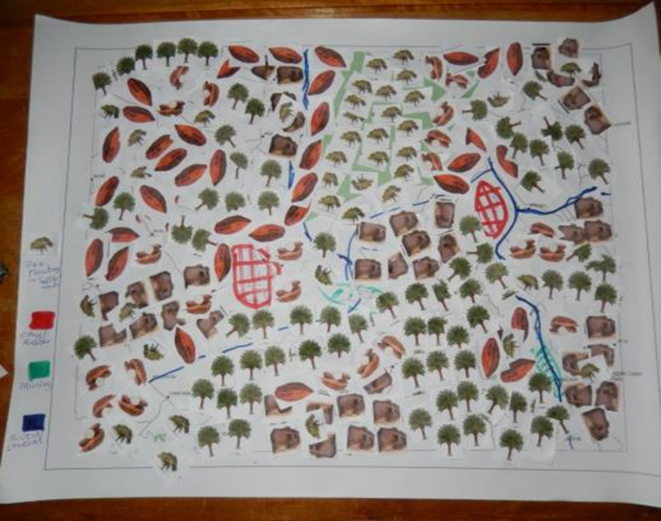 | 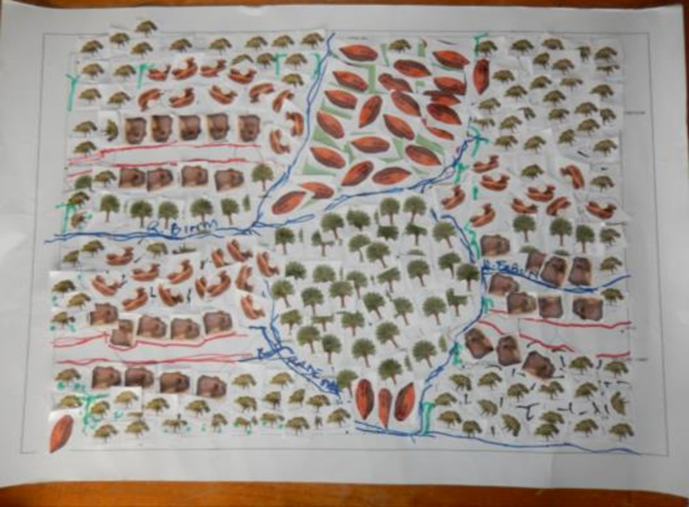 | 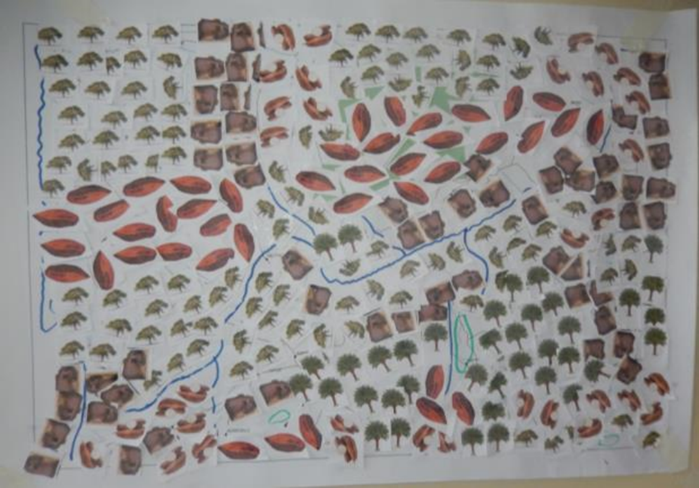 |
| --- | --- | --- |
| Kade rural group | Kade township group | Takorowase group |
|  |  |  |
| 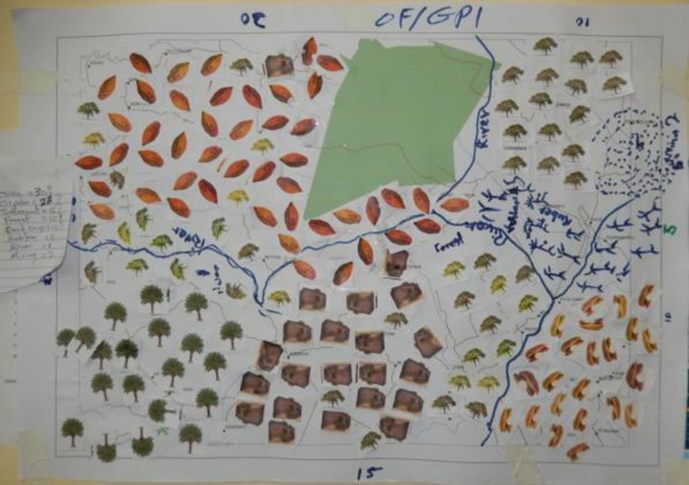 | 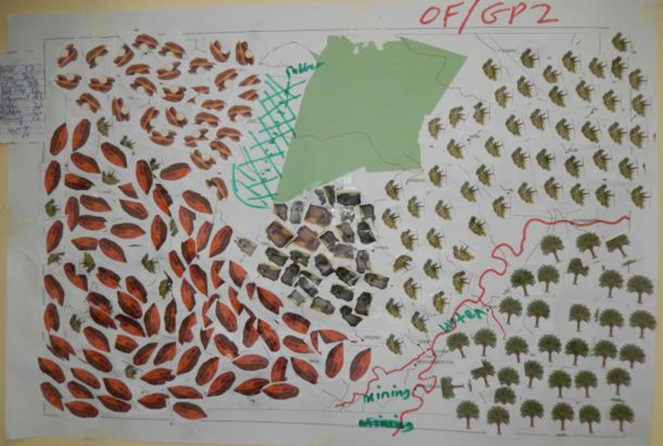 | 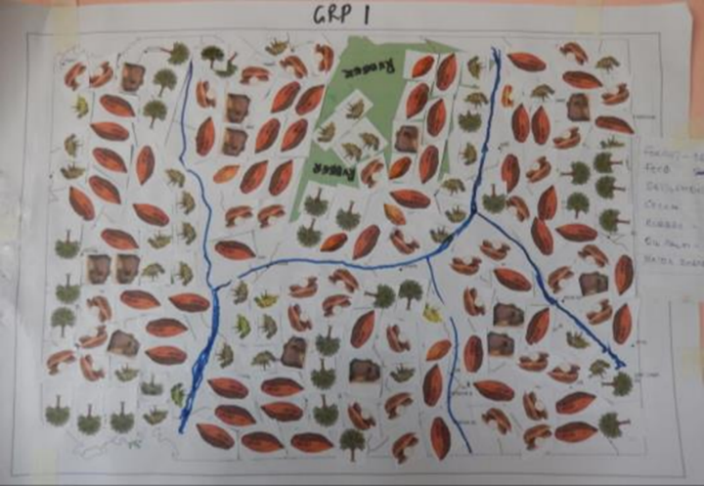 |
| Ofoaso rural group | Ofoaso township group | Abenase rural group |
|  |  |  |
| 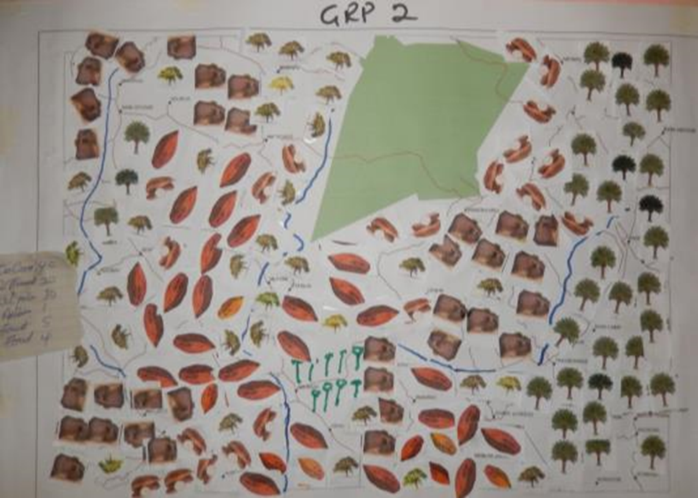 | 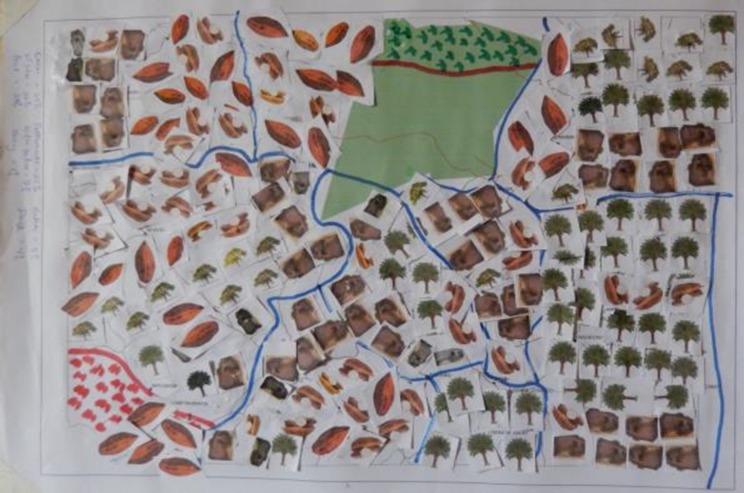 | 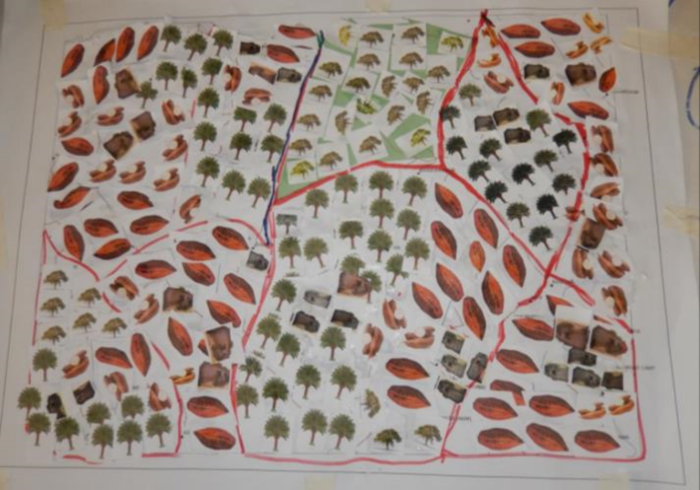 |
| Abenase township group | Akyemansa District Assembly | Kwaebibirem Municipal Assembly |
|  |  |  |

#

# Supplementary material 3: Cluster-specific analysis of farmers’ desired landscapes

The desired landscape of farmers from the *rural, cocoa-dominated area* (Abenase rural) represents a typical traditional rural cocoa landscape with scattered settlements in a landscape marked by a high degree of integration of different land-cover types, including forest patches (segregation score: 0.176). Considering forest land as fertile and perceived to be highly suitable for cocoa, they prefer the forest reserve to be replaced with cocoa farms.

Farmers from the more *urbanized cocoa-dominated landscape* depicted a moderately to highly segregated landscape with clustered settlements. The segregated nature of the desired landscape is reflected in the fact that the land-cover types consist of single blocks of continuous land units. Maps from the Ayirebi and Ofoase townships show a preference for a more consolidated settlement area, surrounded by forest, cocoa and oil palm, with food crops and water in close proximity. Participants motivated the centrality and concentration of built-up areas in the desired landscape with having easy access to farms, and more land available for agricultural activities. They prefer these settlements to be surrounded with tree cover as protection from strong winds. The map from Abenase township, which is closer to the oil palm area, deviates from this picture. It comes closer to those from the oil-palm areas, with a substantial area allocated to oil palm and a more scattered, semi-concentrated settlement pattern. Whereas oil palm is situated in the east in a segregated manner, cocoa appears in a mosaic of settlements, trees, rubber, and food crops. However, the map as a whole shows less integration (segregation score: 0.324) than those from the oil-palm areas and thus occupies a middle position between the oil-palm (average of 0.280) and cocoa-dominated (average of 0.531) areas.

Maps of the desired landscapes from *rural oil-palm dominated areas* (the rural portions of Takorowase and Kade) reveal a high preferred degree of integration (0.285 and 0.154 respectively), with oil palm mainly positioned in the south and southeast. The settlements are scattered over the landscape, surrounded by food-crop areas in relatively small patches. Participants from Takorowase prefer to give up the forest reserve and use it for cocoa farming, planting forest around settlements instead.

The latter also occurs in the map of the highly *urbanized oil-palm area* of Kade township. Having experienced the drawbacks from oil palm expansion, the farmers prefer a 70% forest cover to be positioned as wind breaks close to settlements and food-crop areas. Like the Takorowase participants, they prefer the forest reserve to be replaced with cocoa, arguing that trees from the forest reserve do not generate direct benefits while it provides fertile ground for cocoa farming. The desired landscape is moderately segregated (segregation score of 0.402), somewhat contrasting the preference for integrated landscapes among farmers in the other oil-palm areas.

**Supplementary material 4**

Alignment of participants’ proposed stepwise approach toward achieving their desired landscapes process with the ten principles of an integrated landscape approach (Sayer et al. 2013)

| Features of participants’ stepwise approach | Principles for ILAs by Sayer et al. (2013) |
| --- | --- |
| Sensitization for common landscape concerns | Strengthened stakeholder capacity (10); Common concern entry point (2) |
| Inclusive planning towards desired landscapes | Multifunctionality (4); Multi-stakeholder (5); Negotiated and transparent change logic (6); Resilience (9) |
| Bottom-up laws, policies and guidelines | Clarification of rights and responsibilities (7) |
| Implementation | Multi-stakeholder (5); Negotiated and transparent change logic (6) |
| Engagement with other landscape actors, including traditional authorities based on mutual respect and trust | Common concern entry point (2); Multiple scales (3); Multi-stakeholder (5); Negotiated and transparent change logic (6) |
| Joint monitoring and feedback | Adaptive management & continual learning (1); Participatory and user-friendly monitoring (8) |
